# More Money, More Trust? Target and Observer Differences in the Effectiveness of Financial Overcompensation to Restore Trust

**DOI:** 10.5334/pb.ay

**Published:** 2014-09-16

**Authors:** Tessa Haesevoets, Chris Reinders Folmer, Alain Van Hiel

**Affiliations:** 1 Department of Developmental, Personality, and Social Psychology, Ghent University, Belgium; 2 Erasmus School of Law, Erasmus University, The Netherlands

**Keywords:** trust repair, target-observer differences, financial compensation

## Abstract

Recent research revealed that despite its financial costs, overcompensation is not more effective to restore trust in the perpetrator than equal compensation. In a lab experiment (N = 115), we compared the effects of these compensation sizes for both targets of the compensation and non-involved observers. It was revealed that overcompensation did not yield superior outcomes than equal compensation. Specifically, for targets overcompensation resulted in lower levels of trust than equal compensation, while for observers equal compensation and overcompensation resulted in similar levels of trust. This finding suggests that overcompensation is not a cost-effective trust repair strategy, neither for the targets nor for third party observers. Other implications are discussed as well.

## Introduction

Trust plays a pivotal role in many aspects of our lives, as it represents a necessary ingredient to coordinate and smooth social relationships (Cook, 2001). However, people’s actions and decisions in everyday life offer numerous opportunities for violating trust (Kim, Dirks, Cooper, & Ferrin, 2006), and ample research has shown that such trust breaches may lead to pervasive and persistent negative consequences (e.g., see Lount, Zhong, Sivanathan, & Murnighan, 2008). In many situations, perpetrators try to restore broken trust by the offer of a monetary reimbursement to the victim. Previous research regarding the effectiveness of financial compensation mainly explored compensation that is smaller than or equivalent to the damage suffered. On the basis of these studies (e.g., see Bottom, Daniels, Gibson, & Murnighan, 2002; also see Desmet, De Cremer, & van Dijk, 2011), it was concluded that financial compensation is an effective tool in restoring a victim’s trust. Some scholars have, however, argued that restoring broken trust may ask more from a perpetrator than just exactly restoring the damage (e.g., Kim et al., 2006). When the compensation offered by the perpetrator is of greater value than the financial loss suffered by the victim, we speak of *overcompensation.*

Because overcompensation implies additional costs on top of the expenses of compensation that exactly covers the loss suffered (i.e., *equal compensation*), it is costly for the perpetrator, but at the same time profitable for the victim. From such an economic perspective it is surprising that recent research has shown that overcompensation does not provide any surplus value beyond the level of equal compensation, and that it may even provoke adverse effects. Specifically, overcompensation results in lower levels of trust repair and less favorable perceptions of the perpetrator than equal compensation (Haesevoets, Van Hiel, Reinders Folmer, & De Cremer, 2014). These results are consistent with fairness literature (Engelmann & Strobel, 2004), which has shown that people prefer equal outcomes (cf. equal compensation) above unequal outcomes (cf. the advantageous inequality that results from overcompensation; see Loewenstein, Thompson, & Bazerman, 1989).

Importantly, all previous studies regarding the effectiveness of financial overcompensation merely focused on the target of the compensation, thereby overlooking the potential positive influences that overcompensation may have on non-involved observing parties. Indeed, perpetrators often offer victims an overcompensation, not only to repair their relationship with the victim, but also to avoid reputational damage and to positively influence the ‘general public’, like for instance when a company offers a dissatisfied customer a refund, a coupon, or a product replacement that is worth more than the original purchase price (for a meta-analysis on this matter, see Gelbrich & Roschk, 2011).

In the present research, we investigated whether target-observer differences exist in the effectiveness of financial (over)compensation as a trust repair strategy. Specifically, in line with the results of Haesevoets et al. (2014) we hypothesized that for targets, overcompensation is less effective to repair trust than equal compensation (*Hypothesis 1*). With regard to observers, we formulated two competing hypotheses. According to fairness literature, people evaluate and react not only to the unfairness that they personally experience, but also to the fairness experienced by others (cf. O’Reilly & Aquino, 2011; Zhu, Martens, & Aquino, 2012). Hence, since overcompensation fails to restore equality in outcomes, a first possibility is that – similar to targets ­ overcompensation is also less effective than equal compensation to repair observers’ trust (*Hypothesis 2a*). However, based on the affective forecasting literature (for an overview, see Wilson & Gilbert, 2003), it can be expected that observers are unable to adequately forecast their reactions to overcompensation as they lack direct involvement, and therefore experience it differently than targets. More precisely, it can be argued that people have to experience the advantageous inequality that overcompensation entails themselves for overcompensation to result in lower levels of trust. Following this reasoning, it can be expected that for observers overcompensation has no positive nor negative effects and thus results in similar levels of trust as equal compensation (*Hypothesis 2b*).

## Method

### Participants and Design

One hundred fifteen undergraduate students at Ghent University (75% female, *M*_age_ = 19.05, *SD* = 1.74) participated in an experiment for course credits. We employed a 2 (perspective: target versus observer) ×3 (compensation size: no compensation versus equal compensation versus overcompensation) between-subjects design.

### Procedure

Participants were invited in groups of 12 persons. Upon arrival in the laboratory, participants were informed they would participate in a decision task. It was explained that in this task an allocator and a recipient must decide over the division of a certain amount of money. In the *target conditions*, participants were told that they would play this task in the role of recipient with another player present in the lab who would be assigned to the role of allocator. In the *observer conditions*, participants learnt that they would observe a task that takes place between two other players (i.e., an allocator and a recipient) who were present in the lab.

Before the start of the task, all participants received a budget of €20. To induce a sense of ownership over the money that was going to be divided during the task, both the allocator and the recipient (but not the observer) had to cede €5 of their budget. The allocator would then unilaterally divide this €10. The recipient could not influence this division, and thus had to accept the money offered by the allocator. The trust violation was operationalized by means of an unfair allocation of the resources. That is, the allocator was preprogrammed to allocate €1 to recipient and to keep the remaining €9 for him- or herself.

To examine whether this division is perceived as a transgression by the recipient, we asked participants in the *target conditions* to indicate their satisfaction with the distribution by selecting one of two messages to send to the allocator (i.e., “I am satisfied with how you divided the money” or “I am not satisfied with how you divided the money”). In the *observer conditions*, participants observed the recipient sending the message that he or she was not satisfied with the division. In the *target conditions*, four participants (3.5%) indicated that they were satisfied with the division, and thus did not experience it as a transgression. For these participants the experiment ended at this point. The remaining 111 participants (96.5%) proceeded to the compensation size manipulation.

In the *target conditions*, the participants themselves received or did not receive compensation from the allocator, while in the *observer conditions* the participants observed another person (i.e., the recipient) receiving compensation (or not). In the *no compensation conditions*, the allocator did not give additional money to the recipient. In the *equal compensation conditions*, the allocator gave the recipient €4 extra. Finally, in the *overcompensation conditions*, the allocator offered the recipient an additional €14 (for a more detailed description of this procedure, see Haesevoets et al., 2014).

### Measures

*Trust.* Participants’ trust in the allocator was measured using the six item trust scale of Desmet et al. (2011). A sample item is: “I trust the allocator” (*1* = totally disagree, *7* = totally agree; α = .87).

*Manipulation Checks*. To examine whether the perspective manipulation was successful, we used two items: “To what extent were you the recipient of the compensation?” and “To what extent was another person than you the recipient of the compensation?”. Moreover, to investigate the effectiveness of the compensation size manipulation, participants were asked: “To what extent was the compensation greater than the damage caused by the unequal division of the allocator?”. These three manipulation checks were all measured on a scale form *1* (not at all) to *7* (very much).

## Results

### Manipulation Checks

First, we tested the effectiveness of the perspective manipulation using two one-sample *t* tests. The results for the first manipulation check revealed that for the *target conditions* the sample mean of 4.80 (*SD* = 1.62) significantly deviates from the scale’s theoretical midpoint, *t*(55) = 3.71, *p* < .001. Similarly, for the second manipulation check the analysis revealed that for the *observer conditions* the sample mean of 4.58 (*SD* = 1.61) also significantly differs from the value of 4, *t*(54) = 2.69, *p* = .01. The effectiveness of the compensation size manipulation was subsequently tested using a 2 (perspective) × 3 (compensation size) ANOVA. As expected, participants indicated more often that the compensation was greater than the damage caused by the unequal division in the *overcompensation conditions* (*M* = 6.34, *SD* = 0.97) than in the *equal compensation conditions* (*M* = 2.26, *SD* = 1.41) and the *no compensation conditions* (*M* = 1.44, *SD* = 0.89), *F*(2, 105) = 180.14, *p* < .001, η²*_p_* = .77. A post hoc test (LSD) showed that the mean scores of the three compensation sizes significantly differ from each other (all *p*s < .005). The main effect of perspective and the interaction effect of perspective × compensation size were non-significant, *F*(1, 105) = 0.40, *p* = .531, η²*_p_* = .00 and *F*(2, 105) = 0.22, *p* = .806, η²*_p_* = .00, respectively.

### Trust

**Figure 1:**
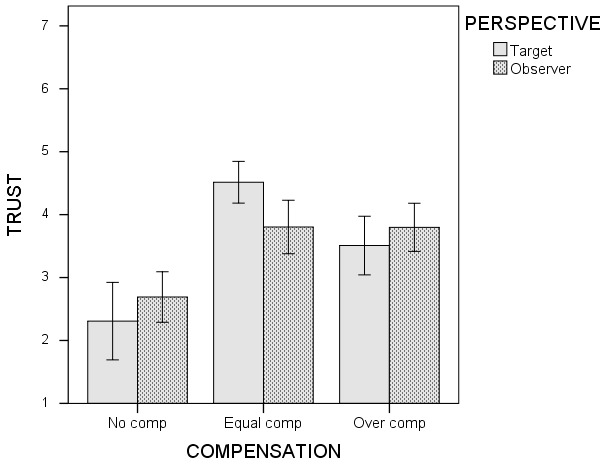
Means trust scores with 95% CI error bars.

A 2 (perspective) × 3 (compensation size) ANOVA on the trust scale showed a non-significant main effect of perspective, *F*(1, 105) = 0.01, *p* = .94, η²*_p_* = .00, a significant main effects of compensation size, *F*(2, 105) = 30.69, *p* < .001, η²*_p_* = .37, and a significant interaction effect of perspective × compensation size, *F*(2, 105) = 4.76, *p* = .011, η²*_p_* = .08. This interaction effect was further explored using planned comparisons. Within both the *target* and the *observer conditions*, a significant effect of compensation size emerged, *F*(2, 105) = 27.19, *p* < .001, η²*_p_* = .34 and *F*(2, 105) = 8.39, *p* < .001, η²*_p_* = .14, respectively. Specifically, for both targets and observers, equal compensation (*M* = 4.51, *SD* = 0.79 and *M* = 3.80, *SD* = 0.96, respectively) and overcompensation (*M* = 3.51, *SD* = 0.97 and *M* = 3.80, *SD* = 0.79, respectively) resulted in higher levels of trust (both *p*s < .001) compared to no compensation (*M* = 2.31, *SD* = 1.02 and *M* = 2.69, *SD* = 0.69, respectively). Further, in line with *Hypothesis 1*, overcompensation is less effective to repair trust (*p* < .001) than equal compensation for targets. Moreover, as predicted by *Hypothesis 2b* (and opposite to the predictions made in the competing *Hypotheses 2a*), for observers no significant difference (*p* = .986) between equal and overcompensation occurred. Figure 1 depicts the means trust scores with 95% CI error bars for each condition.

## Discussion

We replicated the finding of Haesevoets et al. (2014) that despite its considerable costs for the perpetrator and its profitability for the victim, overcompensation has negative effects on the target’s trust in the perpetrator (*Hypothesis 1*). Moreover, we also supplement the current literature by showing that for non-involved observing parties, overcompensation is neither more (nor less) effective than equal compensation to re-establish broken trust (*Hypothesis 2b*). The latter result can possibly be ascribed to the inability of observers to accurately predict their reaction towards overcompensation (see the affective forecasting literature; Wilson & Gilbert, 2003), which seems to align with the idea that people must experience the inequality that results from overcompensation themselves for it to result in a decrease of trust. However, although overcompensation does not entail adverse effects, it also has no additional effect on top of equal compensation in terms of perceived trustworthiness among observing parties. Our results therefore show that overcompensation is not a cost-effective tool to repair broken trust, certainly not for the target of overcompensation, but neither for members of the public.

An important recommendation for further research is to investigate whether these findings also emerge in the context of customer services, as financial compensation is one of the most widely used strategies in service recovery (Davidow, 2003). Previous research in this domain has shown that after a product failure, overcompensation has few, if any, positive effects on the target of the compensation (i.e., the dissatisfied customer; see the meta-analysis of Gelbrich & Roschk, 2011). However, despite the absence of positive effects of overcompensation on targets, companies may generously reimburse dissatisfied customers by providing overcompensation in order to positively influence the general public’s image of the company, like through the creation of positive word-of-mouth which can in turn attract new customers. In this vein, it is surprisingly that, at least to our knowledge, no previous research in the domain of customer services investigated whether overcompensation has indeed positive effects on observing third parties. However, if our finding that overcompensation as a means to resolve a transgression at the interpersonal level entails no positive consequences ­ not for targets nor for observers ­ would also apply to consumer settings, companies should critically assess the use of financial overcompensation as a restoration strategy for a product or service failure.

## Authors’ Note

This research was supported by a research grant from the National Fund for Scientific Research-Flanders (Belgium) under Grant number G095912N. Correspondence concerning this article should be addressed to Tessa Haesevoets, Department of Developmental, Personality, and Social Psychology, Henri Dunantlaan 2, B-9000, Ghent, Belgium. E-mail: Tessa.Haesevoets@UGent.be, Tel.: ++32 (0)9 264 91 41, Fax.: ++32 (0)9 264 64 99.
